# Light-regulated PAS-containing histidine kinases delay gametophore formation in the moss *Physcomitrella patens*

**DOI:** 10.1093/jxb/ery257

**Published:** 2018-08-03

**Authors:** Masashi Ryo, Takafumi Yamashino, Yuji Nomoto, Yuki Goto, Mizuho Ichinose, Kensuke Sato, Mamoru Sugita, Setsuyuki Aoki

**Affiliations:** 1 Graduate School of Information Science, Nagoya University, Furo-cho, Chikusa-ku, Nagoya, Japan; 2 Graduate School of Bioagricultural Sciences, Nagoya University, Furo-cho, Chikusa-ku, Nagoya, Japan; 3 Center for Gene Research, Nagoya University, Furo-cho, Chikusa-ku, Nagoya, Japan; 4 Institute of Transformative Bio-Molecules, Nagoya University, Furo-cho, Chikusa-ku, Nagoya, Japan; 5 Graduate School of Informatics, Nagoya University, Furo-cho, Chikusa-ku, Nagoya, Japan

**Keywords:** Basal land plant, gametophore, histidine kinase, PAS domain, *Physcomitrella patens*, protonema side branch

## Abstract

Two-component systems (TCSs) are signal transduction mechanisms for responding to various environmental stimuli. In angiosperms, TCSs involved in phytohormone signaling have been intensively studied, whereas there are only a few reports on TCSs in basal land plants. The moss *Physcomitrella patens* possesses several histidine kinases (HKs) that are lacking in seed plant genomes. Here, we studied two of these unique HKs, PAS-histidine kinase 1 (PHK1) and its paralog PHK2, both of which have PAS (Per–Arnt–Sim) domains, which are known to show versatile functions such as sensing light or molecular oxygen. We found homologs of PHK1 and PHK2 only in early diverged clades such as bryophytes and lycophytes, but not in seed plants. The PAS sequences of PHK1 and PHK2 are more similar to a subset of bacterial PAS sequences than to any angiosperm PAS sequences. Gene disruption lines that lack either *PHK1* or *PHK2* or both formed gametophores earlier than the wild-type, and consistently, more caulonema side branches were induced in response to light in the disruption lines. Therefore, PHK1 and PHK2 delay the timing of gametophore development, probably by suppressing light-induced caulonema branching. This study provides new insights into the evolution of TCSs in plants.

## Teaser

In the moss *Physcomitrella patens*, PAS-containing histidine kinases regulate the timing of developmental processes of gametophyte generation in response to light, suggesting their functional significance unique to basal land plants.

## Introduction

Two-component systems (TCSs) constitute a major class of signaling pathways broadly observed in various prokaryotic and eukaryotic organisms (except animals) (Stock *et al.*, 2000). In the simplest form of a TCS, a histidine kinase (HK) autophosphorylates upon perception of a stimulus, and this signal is transduced by transferring phosphate to the receiver (REC) domain of a response regulator (RR) protein; the RR then transduces the signal through its output domain, finally modifying transcription of downstream genes. In ‘multistep’ TCSs, which are generally observed in plants, a ‘hybrid’ HK additionally has a REC domain, via which the phosphate is transferred to another TCS component, the histidine-containing phosphotransmitter (HPt), which further relays phosphate to a downstream RR (Pekárová *et al.*, 2016). By having various domains attached to HK as sensor or protein–protein interaction modules, TCSs function as signaling circuitries for responding to various biotic and abiotic environmental stimuli (Mizuno *et al.*, 1996). In plants, TCSs constitute core parts of the signaling pathways for two major phytohormones, cytokinin and ethylene, which elaborately regulate growth and development by integrating different environmental cues (Chang *et al.*, 1993; Hwang and Sheen, 2001; Kakimoto, 2003; Mizuno, 2005; To and Kieber, 2008).

Upon colonization of land from aquatic environments, plants faced a number of novel and tough environmental challenges, such as desiccation, a greater influence of gravity (for lack of buoyancy), a larger range of ambient temperature and strong UV irradiation (Rensing *et al.*, 2008; Shaw *et al.*, 2011). Through adaptation to such harsh environments, plants must have undergone drastic changes in their morphologies and metabolism; moreover, after terrestrialization, plants have diverged and become adapted to a variety of land environments, finally filling many different ecological niches (Raven, 2000; Renzaglia *et al.*, 2000; Shaw *et al.*, 2011). TCSs are likely to have played important roles in adaptation to these changes in the environment (Rensing *et al.*, 2008; Pils and Heyl, 2009; Ishida *et al.*, 2010). Therefore, over the course of evolution, TCSs are also likely to have evolved, probably by acquisition and/or loss of their components, which would have been accompanied by rewiring of their signaling circuitries. A comparison of TCSs between diverse plant lineages could help to shed light on how TCSs have changed and contributed to the evolution of plants. Of particular interest are TCSs of basal land plants, which are descendants of very early diverging lineages of embryophytes (Rensing *et al.*, 2008).

The moss *Physcomtirella patens* belongs to the Bryopsida (mosses), basal land plants that diverged from the lineages leading to extant vascular plants at least 450 million years ago. This moss is an attractive model plant because various molecular biology techniques such as targeted gene disruption are well established (Cove, 2005). Moreover, the entire *P. patens* genome has been sequenced (Rensing *et al.*, 2008). These outstanding features have made *P. patens* a model species of choice for studying growth, physiology and development in terms of plant evolution and diversity (Cove and Knight, 1993; Cove, 2005). In *P. patens*, gene families encoding HKs and RRs are much larger than those found in angiosperm genomes, suggesting a more elaborate use of TCSs in this species (Rensing *et al*, 2008). The model dicot Arabidopsis has genes that encode the following TCS components: 11 HKs (AHKs), six HPts (AHPs) and 23 RRs (ARRs) (To and Kieber, 2008; Pekárová *et al.*, 2016). AHKs can be classified into several subfamilies based on their functions: three cytokinin receptors (AHK2–4), five ethylene receptors (ETR1, ERS, ETR2, EIN4, ERS2), a putative osmosensor (AHK1), a regulator for salt sensitivity (AHK5) and a regulator for female gametophyte development (CKI1) (Kakimoto, 2003; Grefen and Harter, 2004; Desikan *et al.*, 2008; Deng *et al.*, 2010; Pham *et al.*, 2012). *Physcomitrella patens* has a set of ‘classic’ HK sequences that are likely orthologs of cytokinin or ethylene receptors in Arabidopsis (Pils and Heyl, 2009; Ishida *et al.*, 2010; Gruhn *et al.*, 2014; von Schwartzenberg *et al.*, 2016). Notably, the *P. patens* genome also possesses other HK sequences whose multi-domain architectures are found in the genomes of neither seed plants nor green algae (Ishida *et al.*, 2010). Though these unique HKs could serve as clues for gaining insight into an understanding of the evolution of TCSs, to date, no functional analysis of these HKs has been performed.

In the current study, we characterized two *P. patens* genes, *PHK1* and *PHK2*, encoding HKs that contain PAS (Per–Arnt–Sim) domains (Ishida *et al.*, 2010). PAS domains can be found in organisms across all kingdoms of life, and they have various functions such as mediating protein–protein interaction and sensing molecular oxygen, small metabolites and light (Möglich *et al.*, 2009; Henry and Crosson, 2011; Vogt and Schippers, 2015). In angiosperms, PAS-containing proteins show diverse physiological functions such as controlling development, stress adaptation responses and regulation of the circadian clock machinery; these functions of angiosperm PAS-containing proteins largely reflect diversification of their multi-domain architectures (Vogt and Schippers, 2015). Interestingly, no PAS-containing HK was found in the genomes of seed plants or ferns in our survey. Moreover, we demonstrate that the PAS domains of PHK1 and PHK2 are not clustered with any known group of plant PAS-containing proteins. Disruption of *PHK1* and/or *PHK2* induced earlier gametophore formation, probably by promoting caulonema filament branching in response to red light at an earlier stage. We will discuss the functional significance of *PHK1* and *PHK2* from an evolutionary viewpoint.

## Materials and methods

### Plant material and growth conditions

*Physcomitrella patens* ssp. *patens* collected in Gransden Wood (Ashton and Cove, 1977) was maintained in continuous light irradiated by white fluorescence lamps (FL20SS W/18, Toshiba Lighting & Technology Corporation, Yokosuka, Japan; light intensity: ~45 μmol m^−2^ s^−1^) at 25°C. Protonemata were grown on BCD medium supplemented with 1 mM CaCl_2_ or BCDAT medium (BCD medium supplemented with 1 mM CaCl_2_ and 5 mM ammonium tartrate) (Nishiyama *et al.*, 2000). Protonemata were collected every 3–7 d, and were ground with a homogenizer (Physcotron, Microtec, Funabashi, Japan) before they were applied to a new BCDAT agar plate (Nishiyama *et al.*, 2000).

### Cloning of cDNAs for PHK1 and PHK2

The cDNA fragments spanning the entire coding region of *PHK1* or *PHK2* were PCR-amplified using primers PHK1-5′UTR-F2 and PHK1-3′UTR-R2 for *PHK1* and PHK2-5′UTR-Fw and PHK2-3′UTR_Rv for *PHK2* (see Supplementary Table S1 at *JXB* online). Protonemata were grown on BCDAT agar plates for 4 d under continuous light, and then harvested for RNA extraction, which was performed by using the NucleoSpin RNA Plant (Macherey-Nagel, Düren, Germany). The resulting total RNA was used for cDNA synthesis using the ReverTra Ace (Toyobo, Osaka, Japan).

### Phylogenetic analysis

We used a dataset of PAS-containing HKs (see Supplementary Table S2), which were obtained by BLASTP searches (Altschul *et al.*, 1997) against databases, the non-redundant protein dataset from NCBI, Phytozome v12 (https://phytozome.jgi.doe.gov/pz/portal.html) for *Selaginella moellendorffii*, *Sphagnum fallax*, *Marchantia polymorpha*, the *Klebsormidium nitens* NIES-2285 genome project (http://www.plantmorphogenesis.bio.titech.ac.jp/~algae_genome_project/klebsormidium/) for *K. nitens*, using the PHK1 or PHK2 sequence as a query for the phylogenetic tree(s) in Fig. 3 and/or Fig. 4. In addition, we also used a dataset of PAS(/LOV) domain sequences of various known PAS(/LOV)-containing proteins (Supplementary Table S2) in Fig. 4. Amino acid sequences of these datasets were aligned using the ClustalW program (Higgins and Sharp, 1988) and phylogenetic trees were constructed by the maximum likelihood method based on the Jones–Taylor–Thornton (JTT) model (Jones *et al.*, 1992). The numbers at each node represent the percentages for bootstrap support calculated based on 500 bootstrap sampling. Initial tree(s) for the heuristic search were obtained automatically by applying Neighbor-Join and BioNJ algorithms to a matrix of pairwise distances estimated using the JTT model, and then selecting the topology with superior log likelihood value. The analyses involved 10 and 90 amino acid sequences in Figs 3 and 4, respectively. All positions containing gaps and missing data were eliminated. There were 298 and 12 positions in the final datasets for the trees in Figs 3 and 4, respectively. These analyses were conducted in MEGA7 (Molecular Evolutionary Genetics Analysis version 7.0 for bigger datasets) (Kumar, 2016).

### Construction of disruption plasmids

A 1.0-kb genomic region upstream of the *PHK1* gene was PCR-amplified using two primers, PHK1-5′F2 and PHK1-5′R2. The amplified fragment was digested with *Kpn*I and *Apa*I, and cloned into the *Kpn*I–*Apa*I-cleaved pTN182 (a gift from Dr M. Hasebe), resulting in pTN182-phk1_left. A 1.5-kb genomic region downstream of *PHK1* was PCR-amplified using two primers, PHK1-3′F and PHK1-3′R-BamHI. The amplified fragment was digested with *Sma*I and *Bam*HI, and cloned into the *Sma*I–*Bam*HI-cleaved pTN182-phk1_left, resulting in pTN182-phk1. A 1.0-kb genomic region upstream of *PHK2* was PCR-amplified using two primers, PHK2-5′F-SalI and PHK2-5′R(EcoRV). The amplified fragment was digested with *Sal*I and *Eco*RV, and cloned into the *Sal*I–*Eco*RV-cleaved pTN186 (a gift from Dr M. Hasebe), resulting in pTN186-phk2_left. A 1.5-kb genomic region downstream of *PHK2* was PCR-amplified using two primers, PHK2-3′F(EcoRV) and PHK2-3′R-BamHI. The amplified fragment was digested with *Eco*RV and *Bam*HI, and cloned into the *Eco*RV–*Bam*HI-cleaved pTN186-phk2_left, resulting in pTN186-phk2. We used pTN182-phk1 and pTN186-phk2 to disrupt *PHK1* and *PHK2*, respectively, after linearization with *Kpn*I and *Bam*HI treatment for pTN182-phk1 or with *Apa*I and *Bam*HI treatment for pTN186-phk2. The sequences of primers are given in Supplementary Table S1.

### Transformation of *P. patens*

Transformation of *P. patens* was carried out by the polyethylene glycol-mediated method as described previously (Okada *et al.*, 2009). BCDAT agar medium supplemented with 0.5% (w/v) glucose (BCDATG medium) containing either 30 mg l^–1^ hygromycin B (Wako Pure Chemical Industries, Osaka, Japan) or 20 mg l^–1^ G-418 (Wako Pure Chemical Industries) or both was used to select transformants. Integration of foreign DNA into the genomic DNA of stable transformants was confirmed by genomic PCR analyses with primers PHK1_genomic_Fw1 (P1), PHK1_genomic_Rv1.2 (P2), nptII_genomic_Fw2 (P3), Pmcv-R (P4), PHK1_RT-PCR_Fw (P7), and PHK1_RT-PCR_Rv (P8) for *PHK1* disruption, and PHK2_genomic_Fw1 (P11), PHK2_genomic_Rv1 (P12), Pmcv-R (P4), aph4_genomic_Fw2 (P13), PHK2_RT-PCR_Fw (P17), and PHK2_RT-PCR_Rv (P18) for *PHK2* disruption (relative positions of the primers are described in Supplementary Fig. S1). RT-PCR reactions for checking the disruption of *PHK1* and/or *PHK2* were performed using standard procedures with primers PHK1_RT-PCR_Fw and PHK1_RT-PCR_Rv for *PHK1* transcripts, PHK2-5′_RT-PCR_Fw and PHK2-5′_RT-PCR_Rv for *PHK2* transcripts, and PpAct3U1 and PpAct3D1 for actin transcripts as the positive control (Ichikawa *et al.*, 2004). The primer sequences are described in Supplementary Table S1.

### Observation of *P. patens*

For the observation of gametophore formation, small tissue fragments (~1 mm in diameter), taken from protonemata grown on BCDAT medium for 3 d in continuous light, were inoculated and grown for 4 weeks in 16 h light/8 h dark (long day; LD) or 8 h light/16 h dark (short day; SD) on BCDAT medium. The light intensity of the light period was 45 μmol m^−2^ s^−1^. Resulting tissue fragments were observed using a stereomicroscope (SZX16, Olympus, Tokyo, Japan) and images were taken with a digital camera (DP21, Olympus). The lengths of stems and the diameters of protonema tissue portions were measured with ImageJ software (https://imagej.nih.gov). To observe side branching of caulonemata, small tissue fragments (~1 mm in diameter), taken from protonemata grown on BCDAT medium for 3 d in continuous light, were inoculated and grown for 10 d in unilateral dim red light (light intensity: ~8 μmol m^−2^ s^−1^) on BCD medium, and were then irradiated with white (45 μmol m^−2^ s^−1^), blue (30 μmol m^−2^ s^−1^) or red (30 or 45 μmol m^−2^ s^−1^) light for 2 d (Imaizumi *et al.*, 2002; Okano *et al.*, 2009). Blue or red light was irradiated with light emitting diode lamps as the light source (LT20B for blue light and LT20R for red light, Beamtec, Kawaguchi, Japan). To observe bud formation, protonemata were inoculated, cultured, and irradiated with unilateral dim red light in the same way as in the observation of caulonema branching, and then white light (45 μmol m^−2^ s^−1^) was irradiated for 4 d.

### Quantitative real-time reverse transcription PCR

Protonemata were grown on BCDAT agar plates with repeated subcultures every 7 d under LD or SD at least for 18 d before harvesting to extract RNA. Protonemata were collected at the times described in the legends of Figs 2B and 7, 4 d after propagation on new plates. The collected protonemata were ground in liquid nitrogen and total RNA was extracted using the NucleoSpin RNA Plant. cDNA was synthesized using the ReverTra Ace, and quantitative real-time reverse transcription PCR (qRT-PCR) was performed using the Fast SYBR Green Master Mix (Thermo Fisher Scientific, Waltham, MA, USA). The following primer pairs were used: for PHK1, PHK1_qPCR_Fw and PHK1_qPCR_Rv; for PHK2, PHK2_qPCR_Fw and PHK2_qPCR_Rv; for APB1, PpAPB1GSP-F1 and PpAPB1GSP-R1; for APB2, PpAPB2GSP-F1 and PpAPB2GSP-R1; for APB3, PpAPB3GSP-F1 and PpAPB3GSP-R1; for APB4, PpAPB4GSP-F3 and PpAPB4GSP-R3; and for PpTUA1, PpTUA1F and PpTUA1R (Aoyama *et al.*, 2012). The primer sequences are described in Supplementary Table S1.

## Results

### Structural features and expression profiles of two PAS-containing HK genes from *P. patens*

**Fig. 1.**
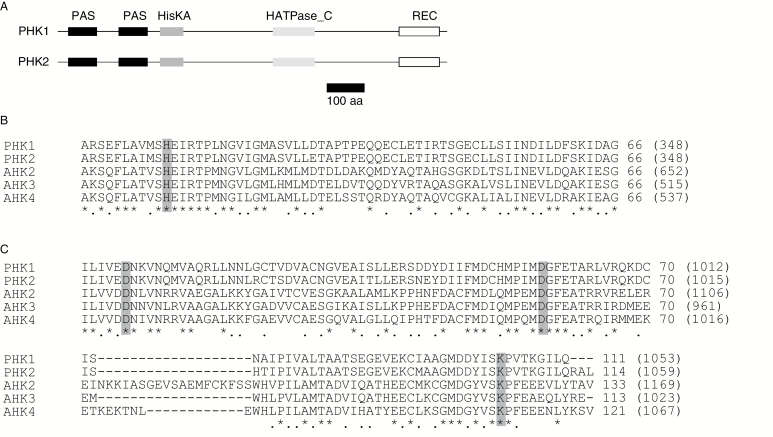
Conserved domains of PHK1 and PHK2. (A) Distribution of conserved domains in PHK1 and PHK2. PAS, HisKA, HATPase_C, and REC domains are indicated by black, dark-gray, light-gray, and open boxes, respectively. (B, C) Amino acid sequence alignments of the HisKA (B) and REC (C) domains of PHK1, PHK2, and AHKs. The conserved histidine residue and aspartic acid–aspartic acid–lysine (DDK) motif, essential for the phosphotransfer activity, are shaded with a gray background. Identical amino acids and amino acids with similar chemical properties are indicated by asterisks and dots, respectively. The number without parentheses shows the last amino acid of each line counted from the first amino acid of each line. The number in parentheses shows the last amino acid of each line counted from the first amino acid of the full-length protein for each sequence. Sequences were aligned using the ClustalW program (Higgins and Sharp, 1988). The HK homologue sequences from Arabidopsis are as follows: AHK2 (AT5G35750), AHK3 (AT1G27320), and AHK4 (AT2G01830).

In a previous paper, Ishida *et al.* (2010) described two PAS-containing HK sequences by searching a former version of the *P. patens* genome database (COSMOSS version 1.1; Rensing *et al.*, 2008). One of them (KEGG entry code: PHYPADRAFT_159715) contains two complete PAS domains, while the other (PHYPADRAFT_125421) has only a partial sequence of a single PAS domain (Ishida *et al.*, 2010). By a BLAST search using these sequences as queries, we obtained two PAS-containing genes, Pp3c16_14810V3.1 and Pp3c27_2730V3.1, corresponding to PHYPADRAFT_159715 and PHYPADRAFT_125421, respectively, in a newer version of the *P. patens* genome database (COSMOSS version 3; Lang *et al.*, 2018). We amplified the coding regions of cDNAs for both genes by reverse-transcription polymerase chain reaction (RT-PCR), determined their sequences, and confirmed that each gene contained two complete PAS domains (Fig. 1A). We named these two cDNAs as *PHK1* (for *PAS-Histidine Kinase 1*; accession number, LC325738; corresponding to Pp3c16_14810V3.1) and *PHK2* (for *PAS Histidine Kinase 2*; accession number, LC325739; corresponding to Pp3c27_2730V3.1). The deduced amino acid sequences of PHK1 and PHK2 are 84% identical to each other and they share identical domain architecture: from the N to the C terminus, two PAS domains (conventionally designated as PAS A and PAS B; Möglich *et al.*, 2009), a histidine kinase (HisKA) domain, a histidine kinase-like ATPase (HATPase_C) domain and a receiver (REC) domain (Fig. 1A). In both proteins, HisKA and REC contain the histidine residue and the aspartic acid–aspartic acid–lysine (DDK) motif (Ueguchi *et al.*, 2001), respectively, that are conserved for phosphate transfer between authentic TCS proteins (Fig. 1B, C).

**Fig. 2.**
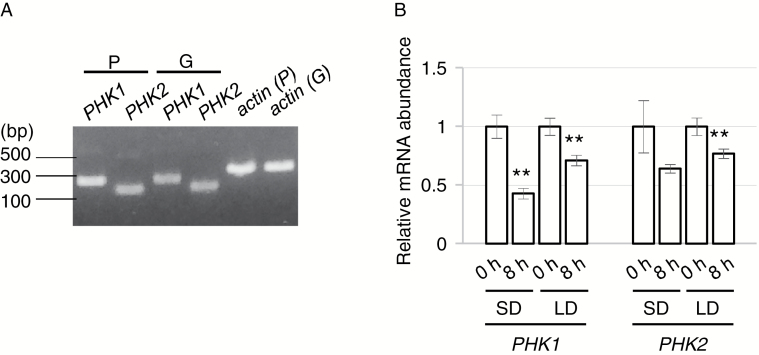
Expression analysis of *PHK1* and *PHK2* genes. (A) Detection of transcripts of *PHK1* and *PHK2* by RT-PCR analysis. Total RNA prepared from protonemata (P) and gametophores (G) was used as template for RT-PCR reactions. Primers for amplification were PHK1_RT-PCR_Fw and PHK2_PHK1_RT-PCR_Rv for *PHK1*, and PHK2_RT-PCR_Fw and PHK2_RT-PCR_Rv for *PHK2*. cDNAs derived from the actin gene (AW698983) were amplified as a positive control with primers PpAct3U1 and PpAct3D1 (Ichikawa *et al.*, 2004). The resulting PCR products were electrophoretically fractionated on an agarose gel (1.0%). (B) Relative transcript levels of *PHK1* and *PHK2* estimated by qRT-PCR. Total RNAs were extracted from protonemata at the end of the dark period (0 h) and 8 h into the light period (8 h) in LD or SD conditions. The α-tubulin gene was used as an internal control. Values are the means ±SD of three technical replicates. Asterisks indicate a statistically significant difference (**P<0.01, *t*-test), compared with the values obtained for the end of the dark period. Three biological replicates were measured and gave similar results. The primer sequences are described in Supplementary Table S1.

Next we examined the expression profiles of *PHK1* and *PHK2*. Transcripts of both genes were detected from both protonema and gametophore tissues by RT-PCR (Fig. 2A). We also examined the relative transcript levels of *PHK1* and *PHK2* by qRT-PCR. For this experiment, we extracted RNA from protonemata grown under a SD (8 h light/16 h dark) or LD (16 h light/8 h dark) photoperiod in order to know whether or not light affects transcription of *PHK1* and/or *PHK2* and, additionally, in order to obtain any evidence that *PHK1* and *PHK2* are under photoperiodic control by comparing the expression profiles between these two conditions. Both genes showed reduced levels of expression at 8 h after the onset of the light period (8 h) compared with the end of the dark period (0 h) in both photoperiodic conditions (Fig. 2B). These results indicate that the *PHK1* and *PHK2* genes are repressed by light, while we could not conclude that these genes are under photoperiodic control because light repression of *PHK* genes was observed in both photoperiodic conditions.

### Phylogenetic analyses

When a BLASTP search (Altschul *et al.*, 1997) was performed against the non-redundant protein dataset from the National Center for Biotechnology Information (NCBI) with the PHK1 or PHK2 sequence as a query, the best and the second best hits (except the *P. patens* PHK1 and PHK2 sequences) were *Selaginella moellendorffii* sequences, XP_002963693 and XP_002974782, which were derived from the same locus (NW_003314264). A BLASTP search performed similarly against the *S. moellendorffii* database within Phytozome (version 12) returned the best hit sequence (405045), which was also derived from this locus. Conversely, a BLASTP search was performed against the *P. patens* genome database (COSMOSS version 3) using the *S. moellendorffii* sequence obtained from Phytozome (405045) as a query, the best and the second best hits were the PHK2 and PHK1 sequences, respectively. Thus, the *S. moellendorffii* sequence (405045) and the *P. patens* PHK2 are reciprocally best hits, indicating that the *S. moellendorffii* sequence is obviously a close homolog to PHK1 and PHK2 (Tatusov *et al.*, 1997; Bork *et al.*, 1998). In the BLASTP search against the NCBI database, there were also many hits with sequences from eubacteria, and most with lower E values are cyanobacterial sequences (the bacterial sequence with the lowest E value (4e-77) is a hypothetical protein (WP007358526.1) of a cyanobacterium *Oscillatoria* sp. PCC 6506). These bacterial sequences showed multi-domain structures similar with those of PHK1 and PHK2, i.e. one or more PAS domains followed by HK-related domains. By searching Phytozome (version 12), we found potential homologs in bryophytes (one from a moss *Sphagnum fallax* and one from a liverwort *Marchantia polymorpha*) in addition to the *S. moellendorffii* sequence. We also found five potential homologs from a charophyte (*Klebsormidium nitens*) through a search on the *K. nitens* NIES-2285 genome project (version 1.0). These sequences, except for the *S. fallax* sequence (Sphfalx0047s0109.1), all have the same domain architectures as PHK1 and PHK2, i.e. PAS-PAS-HisKA-HATPase_C-REC. The *S. fallax* sequence has no HisKA domain and hence seems to be incomplete, but another sequence (Sphfalx0047s0109.2) derived from the same locus as Sphfalx0047s0109.1 shows a HisKA domain sequence; therefore, there is likely to be a complete version of a protein for this gene in *S. fallax* that shows the same domain architecture as *P. patens* PHK1 and PHK2. On Phytozome, we also found sequences from green algae (e.g*. Ostreococcus tauri*, *Chlamydomonas reinhardtii*, *Dunaliella salina* and *Coccomyxa subellipsoidea*) that show similar domain architectures as PHK1 and PHK2; their PAS domains are classified as LOV (light–oxygen–voltage) domains (Djouani-Tahri *et al.*, 2011; Vogt and Schippers, 2015), which comprise a subclass of the PAS domains. By searching ONEKP (Johnson *et al.*, 2012; Matasci *et al.*, 2014; Wickett *et al.*, 2014; Xie *et al.*, 2014), we found two hornwort sequences (UCRN_scaffold_2005453 from *Megaceros tosanus* and FAJB_scaffold_2001990 from *Paraphymatoceros hallii*) and many moss and lycophyte sequences (from species other than *P. patens*, *S. fallax* or *S. moellendorffii*) as potential homologs of PHKs. Interestingly, we found no homologs in angiosperms, gymnosperms, or ferns by searching any databases including ONEKP. Therefore, homologs of PHKs seem to have been lost during evolution in the lineage(s) that lead to extant ferns and seed plants.

**Fig. 3.**
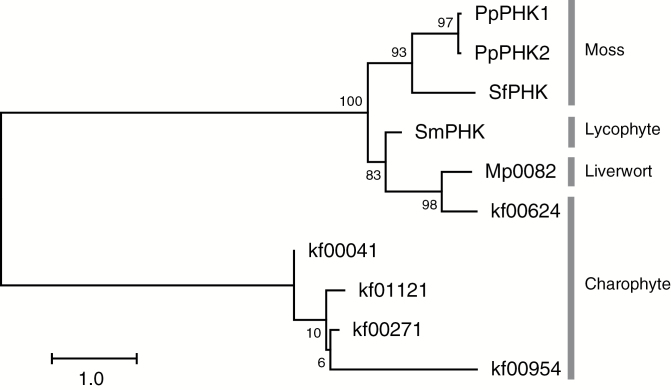
Phylogenetic tree of PHK1 homologs and related sequences in streptophytes. The phylogenetic tree was constructed by the maximum likelihood method using 10 aligned amino acid sequences from streptophyte proteins with the multi-domain structure: two PAS (PAS A and PAS B)-HisKA-HATPase_C-REC domains. Sphfalx0047s0109.1 and Sphfalx0047s0109.2 were combined into the *S. fallax* sequence as SfPHK (see ‘Phylogenetic analyses’ in the Results section). The tree with the highest log likelihood is shown. The tree is drawn to scale with branch lengths measured in the number of substitutions per site. The numbers at each node represent the percentages of bootstrap support, calculated based on 500 bootstrap sampling. For further details, see ‘Phylogenetic analyses’ in the Materials and methods section. Amino acid sequences are as follows: PpPHK1 (LC325738) and PpPHK2 (LC325739) from *P. patens*; SmPHK (405045) from *S. moellendorffii*; SfPHK (Sphfalx0047s0109.1) from *S. fallax*; Mp0082 (Mapoly0082s0006.1) from *M. polymorpha*; kf00624 (kfl00624_0010), kf 00954 (kfl00954_0010), kf01121 (kfl01121_0020), kf00041 (kfl00041_0230), and kf00271 (kfl00271_0210) from *K. nitens.* Also see Supplementary Table S2 for sequence identities. Phylogenetic groupings are indicated on the right.

We constructed a phylogenetic tree by using the above-described sequences from streptophytes with the same domain architectures as PHK1 and PHK2 (PAS-PAS-HisKA-HATPase_C-REC) (Fig. 3). First, PHK1 and PHK2 are connected with each other, next with the *S. fallax* sequence (SfPHK), the complete sequence of which was presumed based on the above-described two sequences Sphfalx0047s0109.1 and Sphfalx0047s0109.2, and then with a cluster consisting of sequences from *S. moellendorffii* (SmPHK), *M*. *polymorpha* (Mp0082), and *K. nitens* (kf00624). These observations indicate that not only the *S. fallax* and *S. moellendorffii* sequences but also the liverwort and charophyte sequences are also homologous to PHK1 and PHK2 (Fig. 3).

**Fig. 4.**
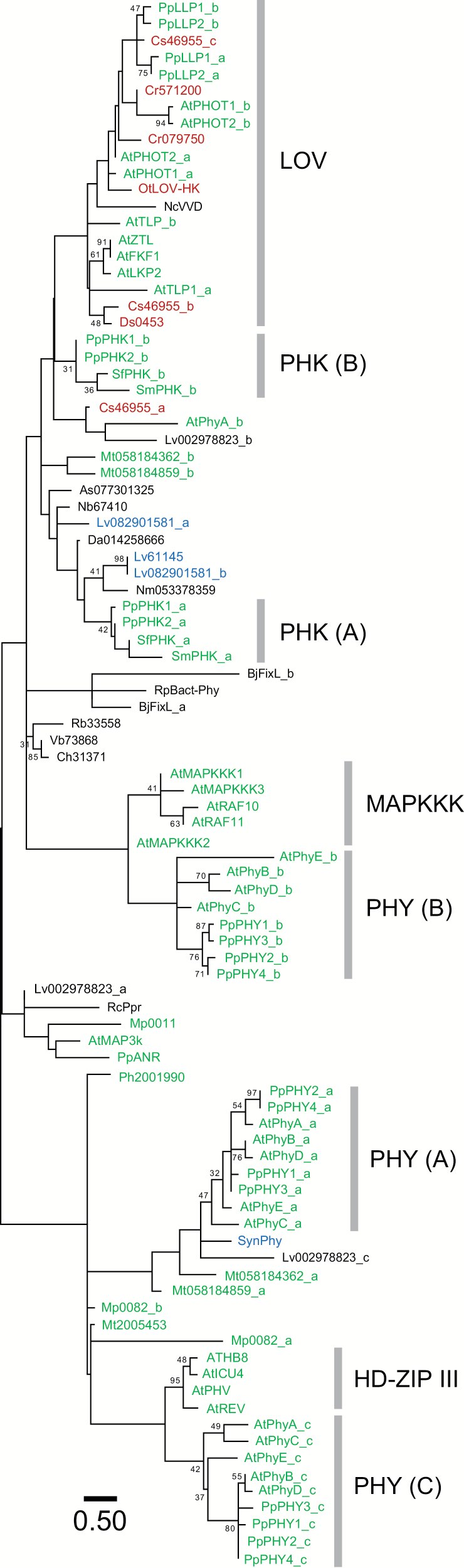
Phylogenetic tree of PAS domains from various PAS-containing proteins. The phylogenetic tree was constructed by the maximum likelihood method using 90 aligned amino acid sequences of PAS domains from PAS-containing proteins of various organisms. Data analysis and representation are the same as in Fig. 3, except that the percentages of bootstrap support are shown only when they are ≥30. Amino acid sequences are as follows: PpPHK1 (LC325738), PpPHK2 (LC325739), PpANR (5IU1), PpPHY1 (AY123146), PpPHY2 (AY123147), PpPHY3 (AY123148), PpPHY4 (AY123145), PpLLP1 (AB576160), and PpLLP2 (AB576161) from *P. patens*; AtPhyA (P14712), AtPhyB (P14713), AtPhyC (P14714), AtPhyD (P42497), AtPhyE (P42498), ATHB8 (Q39123), AtICU4 (Q9ZU11), AtPHV (O04292), AtREV (Q9SE43), AtMAP3K (AEE84700), AtMAPKKK1 (AEE74423), AtMAPKKK2 (AEE74424), AtMAPKKK3 (AEE74425), AtRAF10 (AED95818), AtRAF11 (AEE34716), AtZTL (Q94BT6), AtFKF1 (Q9C9W9), AtLKP2 (Q8W420), AtPHOT1 (O48963), AtPHOT2 (P93025), and AtTLP (O64511) from Arabidopsis; SmPHK (405045) from *S. moellendorffii*; SfPHK (Sphfalx0047s0109.1) from *S. fallax*; Mt2005453 (UCRN_scaffold_2005453) from *M. tosanus*; Ph2001990 (FAJB_scaffold_2001990) from *Paraphymatoceros hallii*; Mp0082 (Mapoly0082s0006.1) and Mp0011 (Mapoly0011s0086.1) from *M. polymorpha*; Cr571200 (Cre13.g571200.t1.1) and Cr079750 (Cre02.g079750.t1.1) from *C. reinhardtii*; Cs46955 (46955) from *C. subellipsoidea* C-169; Ds0453 (Dusal.0453s00005.1) from *D. salina*; OtLOVHK (Ot09g02160) from *O. tauri*; NcVVD (AAK08514) from *Neurospora crassa*; SynPhy (BAA10307) from *Synechocystis* sp. PCC 6803; Mt058184362 (WP_058184362.1) and Mt058184859 (WP_058184859.1) from *Mastigocoleus testarum*; Lv082901581 (WP_082901581.1) and Lv61145 (OAB61145) from *Leptolyngbya valderiana*; Hl088901597 (WP_088901597) and Hi015910083 (WP_015910083) from *Halorubrum lacusprofundi*; AsPAS077301325 (WP_077301325) from *Aquaspirillum* sp. LM1; BjFixL (CAA40143) from *Bradyrhizobium japonicum*; Bm53921 (AAL53921) from *Brucella melitensis* bv. 1 str. 16M; Ch31371 (OYW31371.1) from *Chthoniobacter* sp. 12-60-6; Da014258666 (WP_014258666.1) from *Desulfovibrio africanus*; Lv002978823 (WP_002978823.1) from *Leptospira vanthielii*; Nb67410 (OGW67410) from *Nitrospirae bacterium* RIFCSPLOWO2_01_FULL_62_a7; Nm053378359 (WP_053378359.1) from *Nitrospira moscoviensis*; Rb33558 (OYV33558) from *Rhodospirillales bacterium* 20-64-7; RcPpr (ACJ00586) from *Rhodospirillum centenum* SW; RpBact-Phy (ABI96248) from *Rhodopseudomonas palustris*; Vb73868 (OYW73868.1) from *Verrucomicrobia bacterium* 12-59-8. Also see Supplementary Table S2 for sequence identifiers. According to Möglich *et al.* (2009), multiple PAS domains within one protein are indicated alphabetically from the N to the C terminus, such as PAS_a and PAS_b (representing PAS A and PAS B domains, respectively). PAS domains of Arabidopsis PAS proteins are indicated on the right side with their categories according to Vogt and Schippers (2015). A letter in parentheses indicates the position(s) of PAS within one protein. Sequences of land plants, green algae, cyanobacteria, and other eubacteria are shown in green, red, blue, and black, respectively.

Next, we constructed another phylogenetic tree by using the PAS sequences of various PAS-containing proteins (Fig. 4) that include: (i) PAS (or LOV)-HK proteins in green plants; (ii) various PAS-containing proteins from Arabidopsis and *P. patens*; (iii) bacterial phytochromes; (iv) bacterial PAS-containing proteins that revealed a hit in BLASTP searches by using the full-length sequence of PHK1 or PHK2, or by using one of their PAS sequences as queries; and (v) some other bacterial PAS-containing proteins, in which ligands of their PAS domains are already known. PAS sequences from Arabidopsis formed several clusters, strongly paralleling their domain architectures, which are generally reflected in the division of their physiological functions (Vogt and Schippers, 2015). PAS domains of the *P. patens* proteins other than PHK1 and PHK2 are generally clustered with related PAS domains from Arabidopsis. PAS domains of phytochromes from Arabidopsis and *P. patens* were divided into three clusters (PHY (A), PHY (B) and PHY (C)) according to their positions in the coding regions. LOV domains, which include those of ZTL/FKF1/LTP2 and PHOTs from Arabidopsis, formed a cluster, and LLPs from *P. patens* (Kasahara *et al.*, 2010) also joined this group. The PAS domain of *P. patens* ABSCISIC ACID NON-RESPONSIVE (PpANR) (Stevenson *et al.*, 2016), which has an MAPKKK-like domain as well as a PAS domain, was paired with AtMAP3K, an Arabidopsis MAPKKK protein, though this pair is not grouped with the cluster comprising five PAS domains from other Arabidopsis MAPKKK proteins (MAPKKK). PAS A and PAS B domains of PHKs and their homologs each formed clusters (PHK (A) and PHK (B), respectively, in Fig. 4), and they were not clustered with any of already-known PAS groups, including those from *P. patens*, with significant bootstrap support. PAS domains of above-mentioned bacterial sequences, which showed low E values in the BLAST searches, are closely positioned with PAS A domains of PHKs, although no significant bootstrap support was obtained.

These results suggest that the *PHK1* and *PHK2* genes and their homologs are not closely related to other types of PAS domain-encoding genes in land plants, while there remains a possibility that they are derived from a group of bacterial PAS-HK genes.

### Generation of PHK1 and PHK2 single disruption lines and PHK1 PHK2 double disruption lines

For loss-of-function analysis of *PHK1* and *PHK2*, each gene was disrupted by introducing a drug-resistance gene cassette into its coding region by homologous recombination, generating single gene disruption lines (Supplementary Fig. S1A, B). Double disruption lines, in which both genes are disrupted, were also generated by transforming a *PHK1* single disruption line (*phk1*-13) with the targeting construct for *PHK2*. We selected two independent lines for each single or double disruption (*phk1*-13 and *phk1*-22 for *PHK1* disruption; *phk2*-26 and *phk2*-44 for *PHK2* disruption; *phk1 phk2*-7 and *phk1 phk2*-20 for *PHK1 PHK2* double disruption), and confirmed, using genomic-PCR analysis, that recombination occurred in each of the targeted loci as designed (Supplementary Fig. S1C–J). We also confirmed by RT-PCR analysis that transcripts from the corresponding gene(s) in each of the disruption lines were undetectable (Supplementary Fig. S1K–M).

### Earlier gametophore formation in the disruption lines

**Fig. 5.**
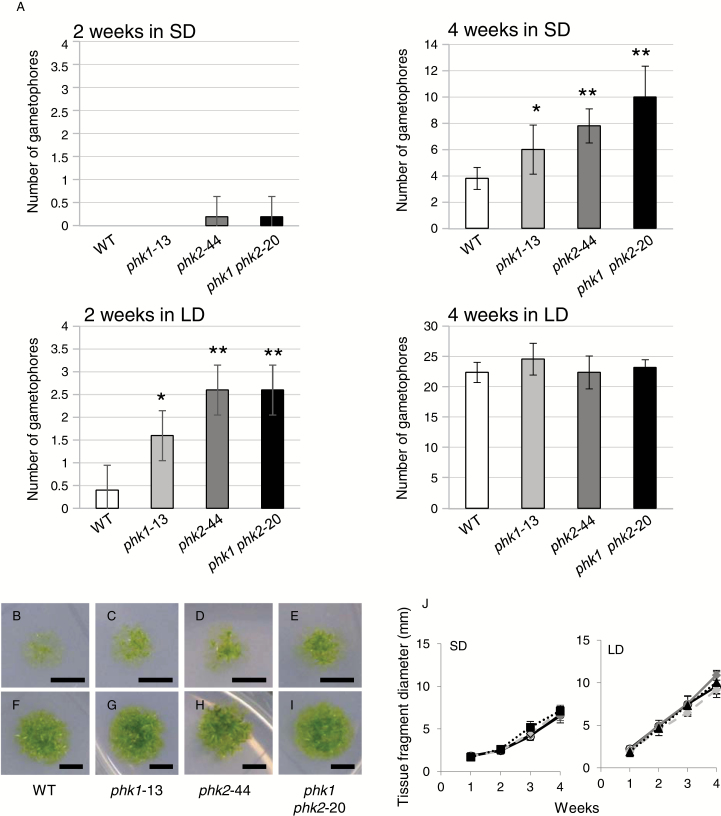
Gametophore formation and protonema tissue growth in *PHK* disruption lines. The number of gametophores that formed in the light–dark cycles was compared between WT and the disruption lines. (A) The number of gametophores that formed after 2 or 4 weeks in SD or LD. Small protonema tissue fragments (~1 mm in diameter) of WT or each of the disruption lines were inoculated and grown on BCDAT medium in each light–dark cycle condition for 2 or 4 weeks, and then the number of emerged gametophores was compared between WT and the disruption lines. The mean numbers ±SD of gametophores per plant obtained from five independent plants are plotted. Asterisks indicate statistically significant differences (*P<0.05, **P<0.01, analysis of variance), compared with the values obtained for WT. (B–I) Representative images of plants grown for 4 weeks are shown for WT (B, F), *PHK1* single (*phk1*-13; C, G), *PHK2* single (*phk2*-44; D, H) and *PHK1 PHK2* double (*phk1 phk2*-20; E, I) disruption lines. Upper (B–E) and lower (F–I) photos represent plants grown in SD and LD, respectively. Scale bar: 5 mm. (J) The sizes of plants were compared between WT and the disruption lines over time in SD or LD. The mean ±SD sizes of two-dimensional protonema tissue portions in diameter from six independent plants grown for periods of time indicated on the horizontal axis are plotted. Open circles with black lines, WT; closed diamonds with gray broken lines, the *PHK1* single disruption; closed triangles with broken lines, *PHK2* single disruption; and closed squares with dotted lines, *PHK1 PHK2* double disruption lines. The diameters were measured using ImageJ software (https://imagej.nih.gov/ij/). Statistical analysis was performed by ANOVA and no significant difference was detected between the lines in each light–dark cycle condition. We obtained similar results in three independent experiments.

To investigate the influence of *PHK1* and/or *PHK2* disruption, we compared gametophore formation between the wild-type (WT) and disruption lines (Fig. 5; Supplementary Fig. S2). A small protonema fragment (~1 mm in diameter) of each line was inoculated and grown on BCDAT medium under different light dark conditions (the neutral day condition (ND; 12 h light/12 h dark), SD, LD or continuous light (LL)), and the number of gametophores was counted. Although we did not obtain data indicating that transcription profiles of *PHK1* and *PHK2* are differentially regulated between different day lengths (Fig. 2B), we still wanted to know whether or not *PHK1* and *PHK2* are involved in photoperiodic control; therefore we used these different photoperiods for this phenotypic analysis. Figure 5A shows gametophore formation at 2 or 4 weeks after inoculation in SD or LD condition. In SD, gametophores were barely observed in all strains within the first 2 weeks after inoculation, and after another 2 weeks had passed, the disruption lines showed more gametophores than WT (Fig. 5A, left, B–E). In LD, on the other hand, all the disruption lines formed more gametophores than WT within 2 weeks after inoculation, but almost no differences were found between these lines after an additional 2 weeks (Fig. 5A, right, F–I). There were no significant differences in the speed of radial growth of protonema filaments (Fig. 5B–J). These results indicate that *PHK1* and *PHK2* repressed gametophore formation in WT. More specifically, PHK1 and PHK2 regulate the developmental timing of gametophore formation but not the final number of gametophores, because similar numbers of gametophores were observed for all lines after 4 weeks in LD (Fig. 5A, bottom right). Consistently, there was no significant difference between the lines in LL (see Supplementary Fig. S3). More gametophores were observed in disruption lines, if an appropriate culture period was chosen (i.e. 2 weeks in LD or 4 weeks in SD), in all the day length conditions tested, except LL. Thus, we could not obtain data indicating that photoperiodic control is involved in the functions of PHK1 and PHK2, consistent with the result of the expression analysis (Fig. 2B). It is probably because the cumulative length of light period in LD (or in LL) for 4 weeks is long enough even for WT to support formation of all the gametophores fully that PHK-dependent delay of gametophore formation was not observed in these conditions (Fig. 5A, right; Supplementary Fig. S3). We confirmed that two independent lines obtained for each single or double disruption showed similar phenotypes (Supplementary Fig. S3). The effects of *PHK1* and *PHK2* disruption were not clearly additive (Fig. 5; 4 weeks in SD and 2 weeks in LD). This suggests the possibility that there is functional divergence between PHK1 and PHK2 or that there is an interaction between PHK1 and PHK2, e.g. they might bind to each other to form a dimer, since HKs are known to function as dimers (Stock *et al.*, 2000).

The stem length and leaf number of gametophores were also compared 4 weeks after inoculation (Supplementary Fig. S2). In SD, the stems of gametophores in all the disruption lines were longer than those in WT (Supplementary Fig. S2C), whereas in LD, only those in the *PHK2* single disruption line were longer than those in WT (Supplementary Fig. S2D). The reason for this difference between SD and LD is probably that most gametophores had performed a longer period of photosynthesis in LD than in SD and they grew to their maximum sizes. There was no significant difference in the ratios of leaf number to stem length between all lines (Supplementary Fig. S2E, F). Additionally, there was no recognizable difference in the basic morphology of gametophores between the lines (Supplementary Fig. S2G, H). The observation that in SD gametophores were longer in disruption lines than in WT is consistent with the idea that *PHK1* and *PHK2* delay the developmental timing of gametophores.

### Earlier formation of side branches of caulonemata in the disruption lines

**Fig. 6.**
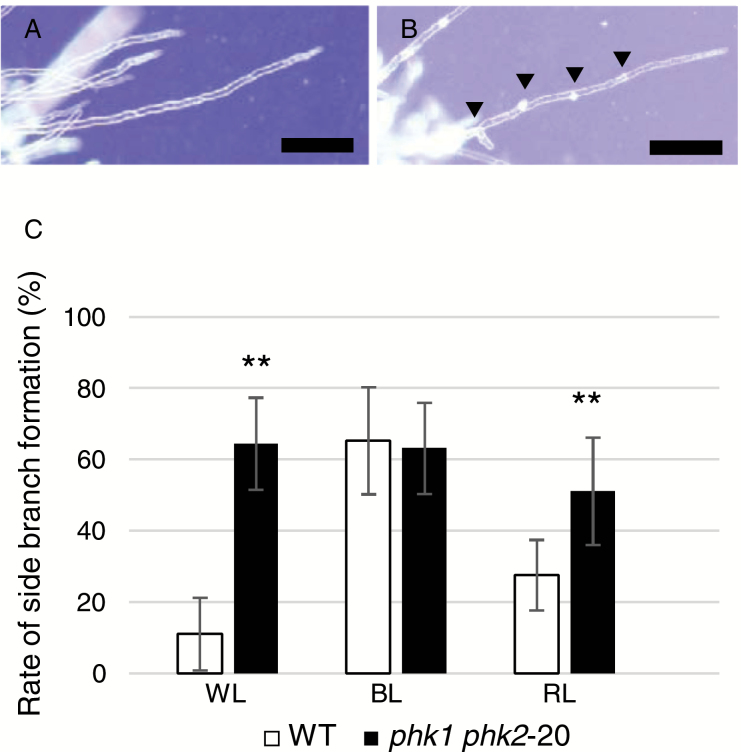
Caulonema side branch formation in *PHK* disruption lines. The number of caulonema side branches induced by light were compared between WT and the double disruption line (*phk1 phk2*-20). Representative images are shown for caulonema filaments of WT (A) and the double disruption line (B) irradiated with white light for 2 d. Bars: 100 μm. Arrowheads indicate side branches (including side branch initial cells). (C) The ratios of side branch formation were compared between WT and the double disruption line. Plotted are the ratios ±SD of cells that formed side branches (including side branch initial cells) within six cells from the filament tip (including the caulonema apical cell, which forms no side branch), obtained from 27–44 independent caulonema filaments. Asterisks indicate statistically significant differences (**P<0.01, *t*-test), compared with the values obtained for WT. Protonemata were inoculated and grown in unilateral dim red light (~8 μmol m^−2^ s^−1^) on BCD medium for 10 d, and then irradiated with white (WL; 45 μmol m^−2^ s^−1^), blue (BL; 30 μmol m^−2^ s^−1^) or red (RL; 30 μmol m^−2^ s^−1^) light for 2 d to induce side branch formation. We obtained similar results for three independent experiments.

We observed early stages of gametophore development in the disruption lines. Gametophores are formed from protonemata through the following developmental processes: (i) chloronema apical cells develop into caulonema cells, (ii) caulonema cells form side branch initial cells, (iii) some of the side branch initial cells develop into buds, and (iv) these buds develop into gametophores (Cove and Knight, 1993, Cove, 2005). First, we compared side branch formation (process (ii)) between WT and the double disruption line. To do this, we grew protonema tissues under unilateral dim red light irradiation (~8 µmol m^−2^ s^−1^) for 10 d, thereby suppressing side branch formation, and then irradiated the cultures with white (45 µmol m^−2^ s^−1^), blue (30 µmol m^−2^ s^−1^), or red (30 µmol m^−2^ s^−1^) light for 2 d in order to induce the synchronous formation of side branch initial cells (Kadota *et al.*, 2000; Okano *et al.*, 2009; Aoyama *et al.*, 2012). We confirmed that caulonema filaments of each line underwent tip growth but formed no side branches during unilateral dim red light irradiation (*n*=13, 8, 10, and 19 for WT, *phk1*-13, *phk2*-44 and *phk1 phk2*-20, respectively). Two days after the onset of light for inducing side branch formation, we counted the number of side branches within six cells from the filament tip (including the caulonema apical cell, which forms no side branch) (Fig. 6; Supplementary Table S3). White, blue, and red light all induced the formation of side branches both in WT and in the double disruption line. In WT, blue light induced formation of more side branches than red light at the same light intensity (Fig. 6C; Supplementary Table S3). Side branch formation was more strongly induced in the disruption line than in WT when irradiated with white or red light (Fig. 6C; Supplementary Table S3). We also observed that single disruption lines of *PHK1* or *PHK2* also showed stronger induction of side branch formation in response to white light than WT (see Supplementary Table S3). These observations indicate that PHK1 and PHK2 repress red light signaling that induces caulonema side branch formation.

### Expression of APB genes in the disruption lines

**Fig. 7.**
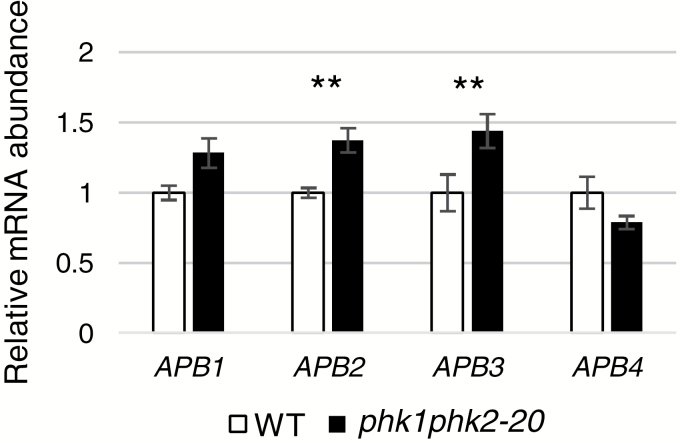
Transcription of the *APB* genes in *PHK* disruption lines. Relative transcript levels of the *APB1*, *APB2*, *APB3*, and *APB4* genes were estimated by qRT-PCR and compared between WT and the double disruption line (*phk1 phk2*-20). Protonemata were cultured in SD and harvested at 5 h into the light period for RNA extraction followed by qRT-PCR. The α-tubulin gene was used as an internal control. Values are the means ±SD of three technical replicates. Asterisks indicate a statistically significant difference (**P<0.01, *t*-test), compared with the values obtained for WT. We obtained similar results for three independent experiments.

Aoyama *et al.* (2012) demonstrated that *P. patens APB* genes (*APB1*~*APB4*), which encode AP2-type transcription factors orthologous to Arabidopsis AINTEGUMENTA, PLETHORA and BABY BOOM (APB) (Shigyo *et al.*, 2006), positively regulate the formation of gametophores. This suggests the possibility that PHK1 and/or PHK2 function through the actions of APBs; therefore, we investigated whether *PHK1* and/or *PHK2* regulate the *APB* genes. We compared the expression levels of the *APB* genes between WT and the double disruption line by qRT-PCR (Fig. 7). The disruption line showed a slightly higher accumulation of mRNA for *APB2* and *APB3* than WT (Fig. 7). This result indicates that PHK1 and/or PHK2 weakly suppress the transcription of *APB2* and *APB3*, which is consistent with the observation of more gametophores in the disruption lines.

## Discussion

### Functions of PHK1 and PHK2 revealed in reverse genetic analyses

Our observations demonstrate that PHK1 and PHK2 delay the timing of gametophore development (Fig. 5A; Supplementary Fig. S3). Moreover, they repress red light signaling that induces caulonema side branch formation (Fig. 6; Supplementary Table S3). A certain proportion (~5%) of caulonema side branch initial cells are known to be destined to become gametophore apical cells (Cove and Knight, 1993), suggesting the possibility that de-repression of light-induced side branch formation due to the absence of PHK1 and PHK2 (Fig. 6) is the basis of earlier gametophore development observed in the disruption lines. (We should keep in mind, however, that there is a possibility that PHK1 and PHK2 also regulate the developmental process (i), i.e. generation of caulonema cells from chloronema apical cells, which could affect the timing of gametophore formation.) While the ratio of caulonema cells forming side branches induced by red light irradiation increased in the disruption line, those induced by blue light irradiation were not significantly different between WT and the disruption line. Therefore, it is supposed that PHK1 and PHK2 are involved in red light signaling, but not in blue light signaling at least in our experimental conditions. It should be noted that blue light induced the formation of more side branches than red light at the same light intensity (Fig. 6C). This is consistent with previous studies showing that side branch formation is dominantly induced by blue light that is mediated by cryptochromes while it is also induced more weakly by red light (Imaizumi *et al.*, 2002; Uenaka *et al.*, 2005). Uenaka *et al.* (2005) suggested the possibility that phytochrome is the photoreceptor responsible for red light induction of side branch formation because red light reception for this response was localized in the nucleus. Further research is required to clarify the molecular details of the process in which PHKs interact with the red light signaling pathway.

The levels of *APB2* and *APB3* expression increased slightly in the double disruption line relative to WT levels, suggesting the possibility that early gametophore formation observed in the *PHK* disruption lines is, at least in part, due to the actions of *APB2* and *APB3*. It is supposed that *APB* genes are indispensable regulators in the formation of gametophore apical cells, but they do not seem to regulate the formation of caulonema side branch initial cells (Aoyama *et al.*, 2012). Therefore, the slight increases of *APB2* and *APB3* suggest that PHK1 and PHK2 also regulate the ratio of bud formation (i.e. the ratio of side branch initial cells that develop into buds to all the side branch initial cells) through APB2 and APB3, in addition to the ratio of caulonema side branch formation. We also attempted to compare the number of buds (process (iii)) between WT and the double disruption line. Three or four days after the onset of white light for the induction of side branch formation, we counted the number of buds in the protonema tissue fragment of WT and the double disruption line. However, we could not obtain reproducible results showing any difference in bud formation between WT and the disruption line (Supplementary Fig. S4). This is probably because the timing of bud formation is not so tightly synchronized by light irradiation as occurs with side branch formation, in addition to the fact that only a small portion of side branch initial cells develop into buds. The data showed relatively large standard deviations (Supplementary Fig. S4), which is probably due to this weakly synchronized and hence more sporadic occurrences of bud formation. Additionally, technical difficulties in identifying buds correctly would be non-negligible factors; sometimes it was not easy to discriminate buds from non-bud side branch cells depending on the angle of the cells for observation, and sometimes gametophore tissues, which should have developed from buds or small gametophores that had been already present when inoculation started, got in the way of observation. In the future, it should be clarified whether PHKs are involved in the regulation of the ratio of bud formation through APBs.

### PHK1 and PHK2 in phylogenetic distribution of PAS-HK proteins

From an evolutionary viewpoint, a remarkable feature of PHK1 and PHK2 is that their eukaryotic homologs are restricted exclusively to relatively early diverged streptophyte lineages, i.e. bryophytes, lycophytes, and possibly a charophyte (Fig. 3). The *S. moellendorffii* and *S. fallax* PAS-HK sequences (405045 and Sphfalx0047s0109.1, respectively) are highly similar and obviously homologous to PHK1 and PHK2 from *P. patens*. Besides, *M. polymorpha* (Mapoly0082s0006.1) and charophyte (kfl00624_0010) sequences are also supposed to be PHK homologs (Fig. 3). Several other PAS-HK proteins, which show identical domain architectures as PHK1 and PHK2, are also found in the charophyte, although they form an independent cluster from PHK1 and PHK2 homologs (Fig. 3). Although PAS-HK proteins were also found in chlorophytes (green algae), a sister group of streptophytes, their PAS domains are further classified into LOV, a subclass of PAS domains. In our tree, consistently, the PAS sequences of green algal proteins (such as Cr571200 and OtLOV-HK) clustered with LOV domains of Arabidopsis LOV proteins such as PHOTs and ZTL (Fig. 4). The LOV domains bind flavin nucleotides and function as a blue-light sensor domain (Herrou and Crosson, 2011; Ito *et al.*, 2012). Djouani-Tahri *et al.* (2011) functionally characterized a LOV-HK protein of the prasinophyte (a group of chlorophytes) *O. tauri*, and revealed that it functions as a blue-light receptor involved in sustaining circadian rhythm under blue light. Thus, the current data suggest that the evolutionary continuity of PHK homologs does not expand to green algae or to fern and seed plant lineages. On the other hand, there were many hits of bacterial PAS-HK sequences in the BLASTP searches using the full-length sequences of PHK1 or PHK2 as a query. Various cofactors that bind to the PAS domains of bacterial PAS-HK proteins are known, such as heme *b* for FixL, FAD for MmoS or NifL, and divalent cations for PhoQ (Henry and Crosson, 2011). Although none of the bacterial proteins found in the BLASTP search or those very closely positioned with PHK1 and PHK2 have, at least to our knowledge, yet been functionally investigated, these cofactor molecules can be clues for investigating the biochemical functions of PHK1 and PHK2 as candidate ligands of their PAS domains.

### Functional significance of PHK1 and PHK2 in light of plant evolution

The phylogenetic distribution of PHK homologs, i.e. presence only in relatively early diverged streptophytes, suggests that PHK homologs function in processes that are absent and no longer important in chlorophytes and relatively recently diverged plants, respectively. In bryophytes, the gametophore (along with the protonema) comprises the haploid gametophyte generation, which is dominant and long-lived in their life cycle. In seed plants, in contrast, the haploid gametophyte generation has a reduced size and only contains a few cells, as in pollen, and it is transient in their life cycle. Moreover, the gametophyte cells in seed plants are internalized in sporophyte tissues, such as the ovule and anther, and are relatively well protected against the environment. Therefore, PHK proteins, developmental regulators of a gametophyte stage in response to a light environment, may have experienced a diminished functional significance during land plant evolution. In this scenario, *PHK* sequences became lost while the gametophyte tissues reduce in size and achieve greater levels of protection against the environment. We found homologs of PHK1 and PHK2 in lycophytes such as *S. moellendorffii*. Lycophytes also show the haploid gametophyte generation that develops independently of the sporophyte, although they show, as seed plants do, a dominant and complex sporophyte generation (Banks, 2009; Banks *et al.*, 2011).

Alternatively, PHK homologs may have been important regulators in the terrestrialization of plants. Gametophores are three-dimensional structures that form sporangia at the top, ultimately scattering spores into the air. Therefore, very early in the evolution of land plants, the emergence of a gametophore or its archetypal structure should have been very important in enabling efficient terrestrialization. The acquisition of *PHK* genes may have played a role in this emergence process as regulators of gametophore development. Extant bryophytes might need PHK proteins because they heavily depend upon an aqueous environment for their reproduction and it is still advantageous to form gametophores for effective scattering of spores. Thus, the phylogenetic distribution of PHK sequences might represent an evolutionary history of the alternation of generations and/or terrestrialization in land plants. In this respect, searching for other regulators present only in early diverged streptophyte lineages may unravel these two highly important processes in plant evolution. Ferns also have a free-living haploid gametophyte generation as with bryophytes and lycophytes, but we could find no fern PAS-HK sequence in any databases. Currently it is unclear whether this is due to absence of PAS-HK homologs or due to a relative paucity of available information for ferns. Elucidating genomes of more diverse species including ferns will add further insights about the origin and evolution of *PHK* genes.

In summary, we demonstrate here: (i) PHK homologs are found only in relatively early diverged lineages in land plants; and (ii) PHKs in *P. patens* delay the timing of developmental processes of the independently living haploid gametophyte generation. These results suggest that PHKs may be involved in evolution of land plants, e.g. in the above-mentioned manners. In the studies of TCSs, a good precedent that relates a particular multi-domain architecture to an important evolutionary process would be the paper by Pils and Heyl (2009); they discuss the possibility that CHASE domain-containing HKs might have been important in terrestrialization of plants, because these CHASE-HKs form a monophyletic group and they are only found in land plant species. PAS-HKs may have been involved in diversification as well as in terrestrialization of land plants, because they are only found in early diverged groups of plants but not in seed plants or ferns. One explanation would be, as described above, that they may have been important in maintenance of the life cycle with an independently living gametophyte generation. In addition to further functional study of PHKs, identification of other TCS components that interact with PHKs will also better clarify functional significance of PHKs in terms of the evolution of TCSs, because it will give us hints of what rewiring occurred in TCSs during plant evolution.

## Supplementary data

Supplementary data are available at JXB online.

Fig. S1. Generation of *PHK* disruption lines.

Fig. S2. The morphology of gametophores in PHK disruption lines.

Fig. S3. Comparison of gametophore formation in different light dark conditions.

Fig. S4. Bud formation in the *PHK1 PHK2* double disruption line.

Table S1. List of primers used in this study.

Table S2. List of protein sequences used in this study.

Table S3. Comparison of side branch formation in response to light irradiation.

## Supplementary Material

Supplementary Figures S1-S4 and Tables S1 S3.pdf
