# Supplementary material for: Light-regulated PAS-containing histidine kinases delay gametophore formation in the moss Physcomitrella patens
Source: J Exp Bot. 2018 Aug 3;69(20):4839–51. doi: 10.1093/jxb/ery257 (PMC6137987; doi:10.1093/jxb/ery257)
Supplement: Supplementary Figures S1-S4 and Tables S1 S3.pdf [file ery257_suppl_supplementary_figures_s1-s4_tables_s1-s3.pdf]

Ryo et al., Figure S1.

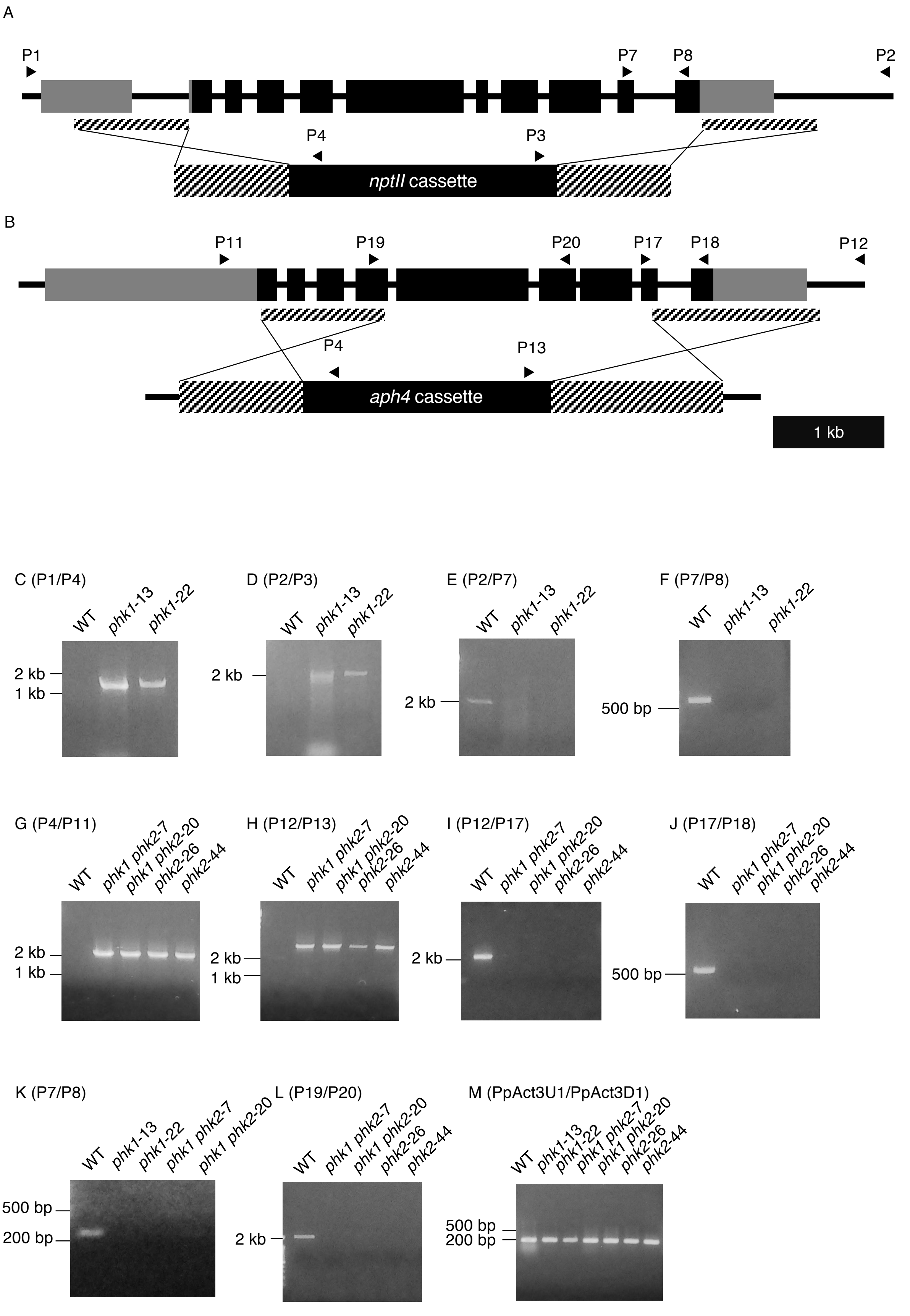

Figure S1. Generation of *PHK* disruption lines. (*Legend appears on following page*.)

Figure S1. Generation of *PHK* disruption lines.

Shown are genomic DNA structures of the *PHK1* (A, top) and *PHK2* (B, top) loci in WT, and their disruption constructs (A and B, bottom). The untranslated regions, coding regions (exons) and introns are indicated by grey rectangles, black rectangles and thick lines, respectively. Homologous recombination target sites are indicated by hatched rectangles. The primers used for genomic PCR analysis are shown as arrowheads. Results of genomic PCR analysis are shown for *PHK1* disruption (C: primer pair, P1/P4; D: P2/P3; E: P2/P7; f: P7/P8) and *PHK2* disruption (G: P4/P11; H: P12/P13; I: P12/P17; J: P17/P18). Bands with predicted sizes were observed for disruption lines, but not for WT (C, D, G, H), when the primer sets designed across a *PHK1* or *PHK2* genomic sequence and a *nptII* or *aph4* gene cassette sequence were used. On the other hand, bands with predicted sizes were observed for WT, but not for the disruption lines (E, F, I, J), when the primer sets designed for coding regions of *PHK1* or *PHK2* were used. Results of RT-PCR analysis are also shown for transcripts of *PHK1* (K: P7/P8), *PHK2* (L: P19/P20) and *actin* (as a control, M: PpAct3U1/PpAct3D1 (Ichikawa et al., 2004) genes. The *PHK1* or *PHK2* transcripts were detected only from WT but not from the disruption lines in which each gene is supposed to be disrupted (K, L), whereas the actin transcripts were detected from all the strains (M). Thus, we verified that *PHK1* and/or *PHK2* were disrupted by homologous recombination as designed. Primer pairs used are indicated on the top of each photo.

Ryo et al., Figure S2.

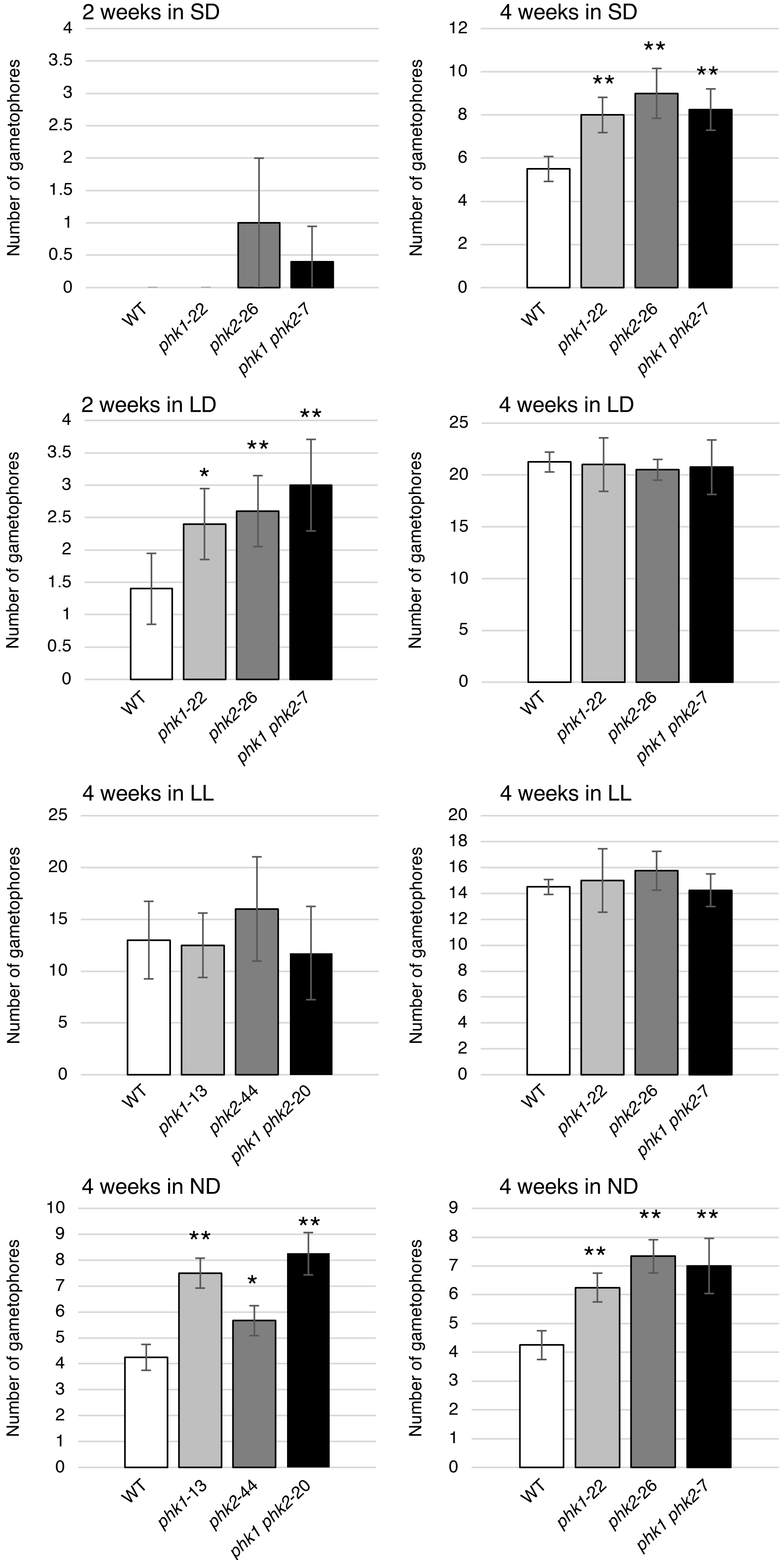

Figure S2. Comparison of gametophore formation in different light dark conditions. (*Legend appears on following page*.)

Figure S2. Comparison of gametophore formation in different light dark conditions.

The numbers of gametophores formed four weeks after inoculation of small protonema tissue fragments in light dark conditions (LL, ND or SD) were counted, and they were compared between WT and the *PHK* disruption lines. Experiments were performed as in Figure 5. The mean numbers (± standard deviations) of gametophores per tissue fragment were calculated from four or five independent fragments. Asterisks indicate statistically significant differences (*, $p < 0.05$; **, $p < 0.01$, ANOVA), compared with the values obtained for WT. We obtained similar results at least two independent experiments.

Ryo et al., Figure S3.

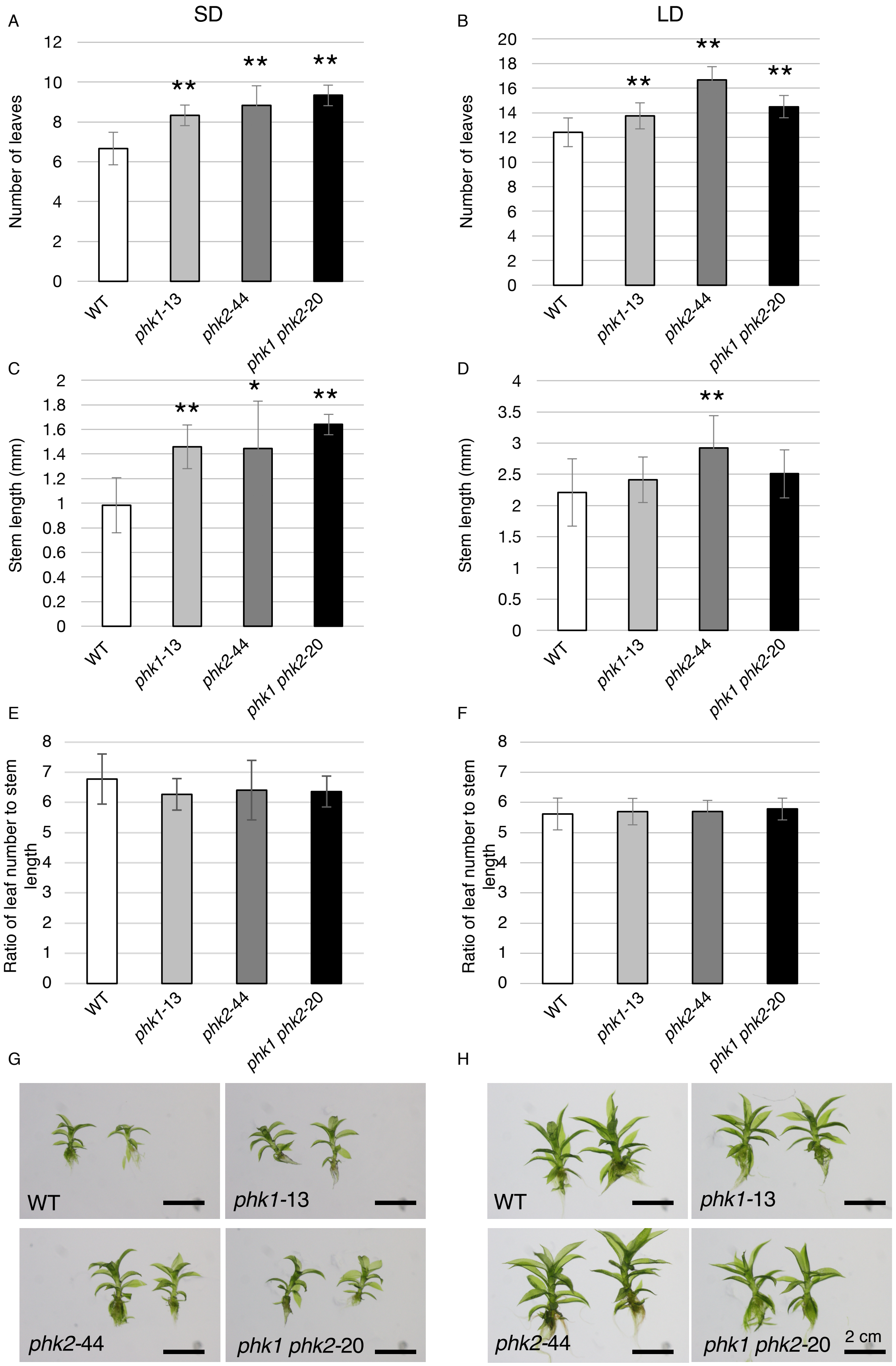

Figure S3. The morphology of gametophores in *PHK* disruption lines. (*Legend appears on following page*.)

Figure S3. The morphology of gametophores in *PHK* disruption lines.

Leaf numbers and stem lengths of gametophores formed on four week-old protonema tissue fragments are compared between WT and the disruption lines (*phk1*-13 for *PHK1* disruption; *phk2*-44 for *PHK2* disruption; *phk1 phk2*-20 for *PHK1 PHK2* double disruption) in SD and LD. Leaf numbers (A and B), stem lengths (C and D) and the ratios of leaf numbers to stem lengths (E and F) of gametophores of WT and the disruption lines in SD (A, C, E) and LD (B, D, F) are shown. The mean values (± standard deviations), each obtained from six gametophores, were plotted. Asterisks indicate statistically significant differences (*, $p < 0.05$; **, $p < 0.01$, ANOVA), compared with the values obtained for WT. We obtained similar results for three independent experiments. Photos of representative gametophores grown in SD (G) and in LD (H) are also shown. Scale bar: 2 cm.

Ryo et al., Figure S4.

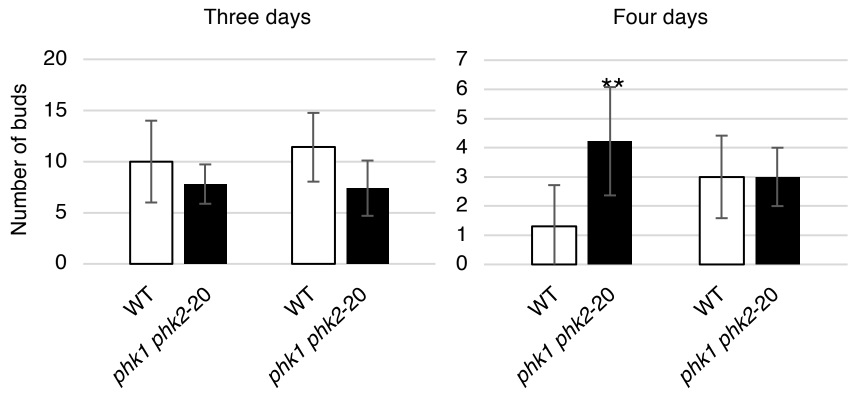

Figure S4. Bud formation in the *PHK1 PHK2* double disruption line.

The number of buds formed on caulonema cells induced by white light were compared between WT and the double disruption line (*phk1 phk2*-20). The number of buds per protonema tissue fragment were compared between WT and the double disruption line after three days (left) or four days (right) of irradiation with white light. The mean values (± standard deviations), each obtained from 10 independent protonema tissue fragments, were plotted. Asterisks indicate a statistically significant difference (**, $p < 0.01$, *t*-test), compared with the value obtained for WT. We present the results of four independent experiments.

Ryo et al., Table S1.

Name	In Figure S1	Sequence (5' to 3')	Reference
Cloning for PHK1 and PHK2			
PHK1-5'UTR-F2		GAATTGTAGAGAGTTAGGTATCTCAAG	
PHK1-3'UTR-R2		GCTATAGAGTGAACAAAACTCGG	
PHK2-5'UTR-Fw		TCGCCACAGGCTGATTCGAACTAC	
PHK2-3'UTR-Rv		GAGTGATTGCGGAGACAGGAGGAG	
Disruption of PHK1 and PHK2			
PHK1-5'F2		AATCGGTACCGATTGAGTGGAACCGAGCG	
PHK1-5'R2		GATGGGGCCCCATCTTGAGATACCTAACTCTCTAC	
PHK1-3'F		GAACCCCGGGGTTCATCAAGGACGGAGGAG	
PHK1-3'R-BamHI		GCGGATCCGCTTTCAAAAAGATCACATCACTGC	
PHK2-5'F-SalI		TGTGGTCGACGTGTTCGTGTGCAACCACG	
PHK2-5'R(EcoRV)		CCGTGATATCCGTAATAGACCC	
PHK2-3'F(EcoRV)		GAATATGATATCATTTTCATGGACTGTC	
PHK2-3'R-BamHI		GCGGATCCCGAAGCTGAATTCGTATTAGGC	
Genomic PCR for PHKs disruption			
PHK1_genomic_Fw1	P1	CTATATGATGACCCGCAGTCAA	
PHK1_genomic_Rv1.2	P2	AAATTCCAACTATGAAATTACAACC	
PHK2_genomic_Fw1	P11	TAAGCCTTCAGGTCGCCACA	
PHK2_genomic_Rv1	P12	GATGCATCTGTCAAGCCGGT	
nptII_genomic_Fw2	P3	ATCGCCGCTCCCGATTCGCA	
aph4_genomic_Fw2	P13	GCAGGGTCGATGCGACGCAA	
Pmcv-R	P4	GAGGAAGGGTCTTGCGAAGGATAGTG	Aoyama et al., 2012
PHK1_RT-PCR_Fw	P7	CTCAGCGACTGCTCAACAATTTGG	
PHK1_RT-PCR_Rv	P8	CAGCTATGCACTTCTCCACCTCTC	
PHK2_RT-PCR_Fw	P17	CAGCGACTGCTCAACAATTTGAGG	
PHK2_RT-PCR_Rv	P18	CGAGATGCAATCCTTCTGCCGCAC	
RT-PCR			
PHK1_RT-PCR_Fw	P7	CTCAGCGACTGCTCAACAATTTGG	
PHK1_RT-PCR_Rv	P8	CAGCTATGCACTTCTCCACCTCTC	
PHK2 -5'_RT-PCR_Fw	P19	AAGAGGAGCAAGCTCGAC	
PHK2-5'_RT-PCR_Rv	P20	GTATTGGAGGTCGTAGGAGG	
PpAct3U1		CGGAGAGGAAGTACAGTGTGTGGA	Ichikawa et al., 2004
PpAct3D1		ACCAGCCGTTAGAATTGAGCCCAG	Ichikawa et al., 2004
Quantitative Real Time-PCR			
PHK1_qRCR_f1		CTTTGCGTTCGCCTTTCC	
PHK1_qPCR_r1		CACAACCGACCGCTCTACAA	
PHK2_qRCR_f1		CATTCAGTTTCCATTCGACAGC	
PHK2_qPCR_r1		AAAGCTCCACTACTGATCGCTCTAC	
PpAPB1GSP-F1		CCATCCACGCGGTTGATAGT	Aoyama et al., 2012
PpAPB1GSP-R1		TCACAGGATCACGAAGGACAAA	Aoyama et al., 2012
PpAPB2GSP-F1		CGGTCCGCGGGAAAG	Aoyama et al., 2012
PpAPB2GSP-R1		TGGGACTGGGAACTCGTCAT	Aoyama et al., 2012
PpAPB3GSP-F1		GGCGAATTGTCGGCATCT	Aoyama et al., 2012
PpAPB3GSP-R1		TCTGCGCCTGACCTGAGTACT	Aoyama et al., 2012
PpAPB4GSP-F3		CGTGCGCTAGTCTGTGCTAGTG	Aoyama et al., 2012
PpAPB4GSP-R3		CCACCTGGATTGGATGCAA	Aoyama et al., 2012
PpTUA1F		CGTAGGAGGGACCAGTTTGG	Aoyama et al., 2012
PpTUA1R		TGCATTCATCCCCGAGTCA	Aoyama et al., 2012

Table S1. List of primers used in this study.

Ryo et al., Table S2.

Name in figure	Accession #	Protein name
Arabidopsis thaliana		
AtPhyA	P14712	Phytochrome A
AtPhyB	P14713	Phytochrome B
AtPhyC	P14714	Phytochrome C
AtPhyD	P42497	Phytochrome D
AtPhyE	P42498	Phytochrome E
ATHB8	Q39123	ATHB-8
AtICU4	Q9ZU11	ATHB-15
AtPHV	O04292	ATHB-9
AtREV	Q9SE43	REVOLUTA
AtMAP3K	AEE84700	MAP3Kδ4
AtMAPKKK1	AEE74423	Putative MAPKKK1
AtMAPKKK2	AEE74424	Putative MAPKKK2
AtMAPKKK3	AEE74425	Putative MAPKKK3
AtRAF10	AED95818	RAF10
AtRAF11	AEE34716	RAF11
AtZTL	Q94BT6	Zeitlupe
AtFKF1	Q9C9W9	FKF1
AtLKP2	Q8W420	LKP2
AtPHOT1	O48963	Phototropin-1
AtPHOT2	P93025	Phototropin-2
AtTLP	O64511	TLP1
*Selaginella moellendorffii**1		
SmPHK	405045	SmPAS-HK
Physcomitrella patens		
PpPHK1	LC325738	PpPAS-HK1
PpPHK2	LC325739	PpPAS-HK2
PpPHY1	AY123146	Phypa;PHY;1
PpPHY2	AY123147	Phypa;PHY;2
PpPHY3	AY123148	Phypa;PHY;3
PpPHY4	AY123145	Phypa;PHY;4
PpANR	5IU1	
PpLLP1	AB576160	LOV/LOV protein1
PpLLP2	AB576161	LOV/LOV protein2
PpPHOTA1	AB163420	Phototropin
PpPHOTA2	AB163421	Phototropin
PpPHOTB1	AB163422	Phototropin
PpPHOTB2	AB163423	Phototropin
*Sphagnum fallax**1		
SfPHK	Sphfalx0047s0109.1	SfPAS-HK
*Marchantia polymorpha**1		
Mp0082	Mapoly0082s0006.1	
Mp0011	Mapoly0011s0086.1	
*Megaceros tosanus**2		
Mt2005453	UCRN_scaffold_2005453 Megaceros_tosanus	
*Paraphymatoceros hallii**2		
Ph2001990	FAJB_scaffold_2001990 Paraphymatoceros_hallii	
*Klebsormidium flaccidum**3		
Kf00624	kfl00624_0010	
Kf00954	kfl00954_0010	
Kf01121	kfl01121_0020	
Kf00003	kfl00003_0010	
Kf00041	kfl00041_0230	
Kf00271	kfl00271_0210	
*Chlamydomonas reinhardtii**1		
Cr571200	Cre13.g571200.t1.1	
Cr079750	Cre02.g079750.t1.1	
*Coccomyxa subellipsoidea C-169**1		
Cs46955	46955	

Table S2. List of protein sequences used in this study. (continued on the next page)

Ryo et al., Table S2.

Name in figure	Accession #	Protein name
*Dunaliella salina**1		
Ds0453	Dusal.0453s00005.1	
Ostreococcus tauri		
OtLOVHK	Ot09g02160	LOV-HK
Neurospora crassa		
NcVVD	AAK08514	VVD
Synechocystis sp. PCC 6803		
SynPhy	BAA10307	Phytochrome
Mastigocoleus testarum		
Mt058184362	WP_058184362.1	
Mt058184859	WP_058184859.1	
Leptolyngbya valderiana		
Lv082901581	WP_082901581.1	
Lv61145	OAB61145	
Halorubrum lacusprofundi		
Hl088901597	WP_088901597	
Hi015910083	WP_015910083	
Aquaspirillum sp. LM1		
AsPAS077301325	WP_077301325	
Bradyrhizobium japonicum		
BjFixL	CAA40143	FixL
Brucella melitensis bv. 1 str. 16M		
Bm53921	AAL53921	
Chthoniobacter sp. 12-60-6		
Ch31371	OYW31371.1	
Desulfovibrio africanus		
Da014258666	WP_014258666.1	
Leptospira vanthielii		
Lv002978823	WP_002978823.1	
Nitrospirae bacterium RIFCSPLOWO2_01_FULL_62_a7		
Nb67410	OGW67410	
Nitrospira moscoviensis		
Nm053378359	WP_053378359.1	
Rhodospirillales bacterium 20-64-7		
Rb33558	OYV33558	
Rhodospirillum centenum SW (Rhodocista centenaria SW)		
RcPpr	ACJ00586	Bacteriophytochrome Ppr
Rhodopseudomonas palustris		
RpBact-Phy	ABI96248	Bacteriophytochrome
Verrucomicrobia bacterium 12-59-8		
Vb73868	OYW73868.1	
Name in figure	Accession #	Protein name

Table S2. List of protein sequences used in this study (continued from the previous page) . Accession numbers refer to those in GenBank except the following: *1, Phytozome v12 (https://phytozome.jgi.doe.gov/pz/portal.html); *2, OneKP (https://sites.google.com/a/ualberta.ca/onekp/); *3, *Klebsormidium nitens* NIES-2285 genome project (http://www.plantmorphogenesis.bio.titech.ac.jp/~algae_genome_project/klebsormidium/)

Ryo et al., Table S3.

	White light (45)			
	average (%)	s.d.	ANOVA	*t*-test
WT	16.8	14.9	$p < 0.01$	-
phk1-13	70	17.2		$p < 0.01$
phk1-22	54.1	14.5		$p < 0.01$
phk2-26	52.5	11.5		$p < 0.01$
phk2-44	59.4	13.9		$p < 0.01$
phk1 phk2-7	54.5	13.5		$p < 0.01$
phk1 phk2-20	64.4	12.9		$p < 0.01$

	BL (30)			
	average (%)	s.d.	ANOVA	*t*-test
WT	53.8	15.2	n.d.	-
phk1 phk2-7	58.3	14.3		n.d.
phk1 phk2-20	58.9	16.7		n.d.

	RL (30)			
	average (%)	s.d.	ANOVA	*t*-test
WT	25.2	12.6	$p < 0.01$	-
phk1 phk2-7	52.7	13.1		$p < 0.01$
phk1 phk2-20	48.6	13.5		$p < 0.01$

Table S3. Comparison of side branch formation in response to light irradiation. The numbers of caulonema side branches induced by light were counted, and the ratios of side branch formation were compared as in Figure 6. White light (top table; 45 μmol m^{-2} sec^{-1}), blue light (middle table; 30 μmol m^{-2} sec^{-1}) and red light (bottom table; 30 μmol m^{-2} sec^{-1}) were used for light irradiation. Shown are the average ratios (average (%)) of cells that formed side branches (including side branch initial cells) within six cells from the filament tip (including the caulonema apical cell), obtained from 24 to 44 independent caulonema filaments. We obtained similar results for at least two independent experiments. Statistically significant differences ($p < 0.01$) were detected with ANOVA and *t*-test between WT and disruption lines when white light or red light was used. s.d., standard deviation. n.d., no difference.
